# A randomized trial examining the effect of predictive analytics and tailored interventions on the cost of care

**DOI:** 10.1038/s41746-021-00449-w

**Published:** 2021-06-03

**Authors:** Mariana Nikolova-Simons, Sara Bersche Golas, Jorn op den Buijs, Ramya S. Palacholla, Gary Garberg, Allison Orenstein, Joseph Kvedar

**Affiliations:** 1 Philips Research, Eindhoven, The Netherlands; 2 Partners Connected Health Innovation, Partners HealthCare, Boston, MA USA; 3 Massachusetts General Hospital, Boston, MA USA; 4 Harvard Medical School, Boston, MA USA; 5 Tufts University School of Medicine, Boston, MA USA; 6 Partners HealthCare at Home, Waltham, MA USA; 7 Philips Population Health Management, Framingham, MA USA

**Keywords:** Randomized controlled trials, Outcomes research, Public health

## Abstract

This two-arm randomized controlled trial evaluated the impact of a Stepped-Care intervention (predictive analytics combined with tailored interventions) on the healthcare costs of older adults using a Personal Emergency Response System (PERS). A total of 370 patients aged 65 and over with healthcare costs in the middle segment of the cost pyramid for the fiscal year prior to their enrollment were enrolled for the study. During a 180-day intervention period, control group (CG) received standard care, while intervention group (IG) received the Stepped-Care intervention. The IG had 31% lower annualized inpatient cost per patient compared with the CG (3.7 K, $8.1 K vs. $11.8 K, *p* = 0.02). Both groups had similar annualized outpatient costs per patient ($6.1 K vs. $5.8 K, *p* = 0.10). The annualized total cost reduction per patient in the IG vs. CG was 20% (3.5 K, $17.7 K vs. $14.2 K, *p* = 0.04). Predictive analytics coupled with tailored interventions has great potential to reduce healthcare costs in older adults, thereby supporting population health management in home or community settings.

## Introduction

COVID-19 has profoundly changed health systems around the world and highlighted the importance of accelerating digital medicine^1^. In particular, telehealth use has surged in response to the COVID-19 pandemic due to its essential role in mitigating increased burden placed on health systems by providing quality care while safeguarding patients’ and healthcare providers’ own health and safety^2^. The US telehealth market is estimated to experience a staggering seven-fold growth by 2025, resulting in a five-year compound annual growth rate of 38%. By the end of 2020, the telehealth market is projected to have grown by 64%^3^. Across the telehealth market segments, virtual visits, and remote patient monitoring will propel the overall market, followed by mHealth and personal emergency response systems (PERS).

The World Health Organization’s Global Strategy on Digital Health^4^ has pushed healthcare organizations (HCOs) to evaluate their ability to broadly deploy telehealth-based population health programs to ensure safe, equitable, and meaningful access to healthcare services. One of the pioneers in telehealth is Mass General Brigham (MGB, formerly Partners HealthCare System), an integrated delivery network located in Massachusetts. It is comprised of two large academic medical centers as well as community hospitals. Partners HealthCare at Home (PHH), is an entity within MGB that deploys population health management programs leveraging many connected health technologies, including PERS.

The PERS is designed to help older adults to live independently in their homes by providing access to immediate assistance in case of medical emergencies that could lead to costly emergency department (ED) visits and hospitalizations. PERS consists of a help button worn as a necklace or bracelet that a patient may press at any time to connect to a 24/7 emergency response center. A response center associate obtains information about the situation and contacts either an informal responder (e.g., neighbor, a family member) or an emergency medical service (EMS, e.g., ambulance, police, or fire department) based on the patient’s specific situation, and then follows up to confirm that help has arrived. Recently, PERS services have been improved with a predictive model that utilizes PERS data to identify patients at risk of ED transports^5^. Such risk predictions can support healthcare providers to proactively intervene and potentially prevent unnecessary healthcare utilization and costs in older patients. That is why such a predictive model was embedded in a Stepped-Care intervention as a first step, triggering nurse-driven interventions as a second step. To evaluate the impact of the Stepped-Care intervention on the healthcare utilization and costs of older patients using PERS we implemented a randomized controlled trial with 370 patients^6,7^.

Many HCOs target costly interventions to their most expensive patients, e.g., the top 5% segment of the cost pyramid, who incur an outsized portion of healthcare costs^8,9^. Although these programs have demonstrated improvement in clinical outcomes, they have not always yielded the necessary cost savings^10–12^. Further, our previously published longitudinal retrospective study of healthcare costs of an older population has shown that the middle segment was persistently the costliest segment through all 5 years with the highest increase in annualized costs compared with the other segments^13^. Informed by these key findings, this study focuses on patients in the middle segment of the cost pyramid.

The study objective was to evaluate the impact of a Stepped-Care intervention on the healthcare utilization and healthcare costs of older patients using PERS. This paper focuses on evaluating healthcare costs associated with healthcare utilization of patients in the middle segment of the cost pyramid. The impact of the Stepped-Care intervention on healthcare utilization outcomes was presented in a separate manuscript^7^ and summarized in the results section of this paper.

We were not able to combine clinical and financial outcomes in one paper as the cost data were available for analysis 8 months after the clinical outcomes data.

## Results

### Participants flow

**Fig. 1:**
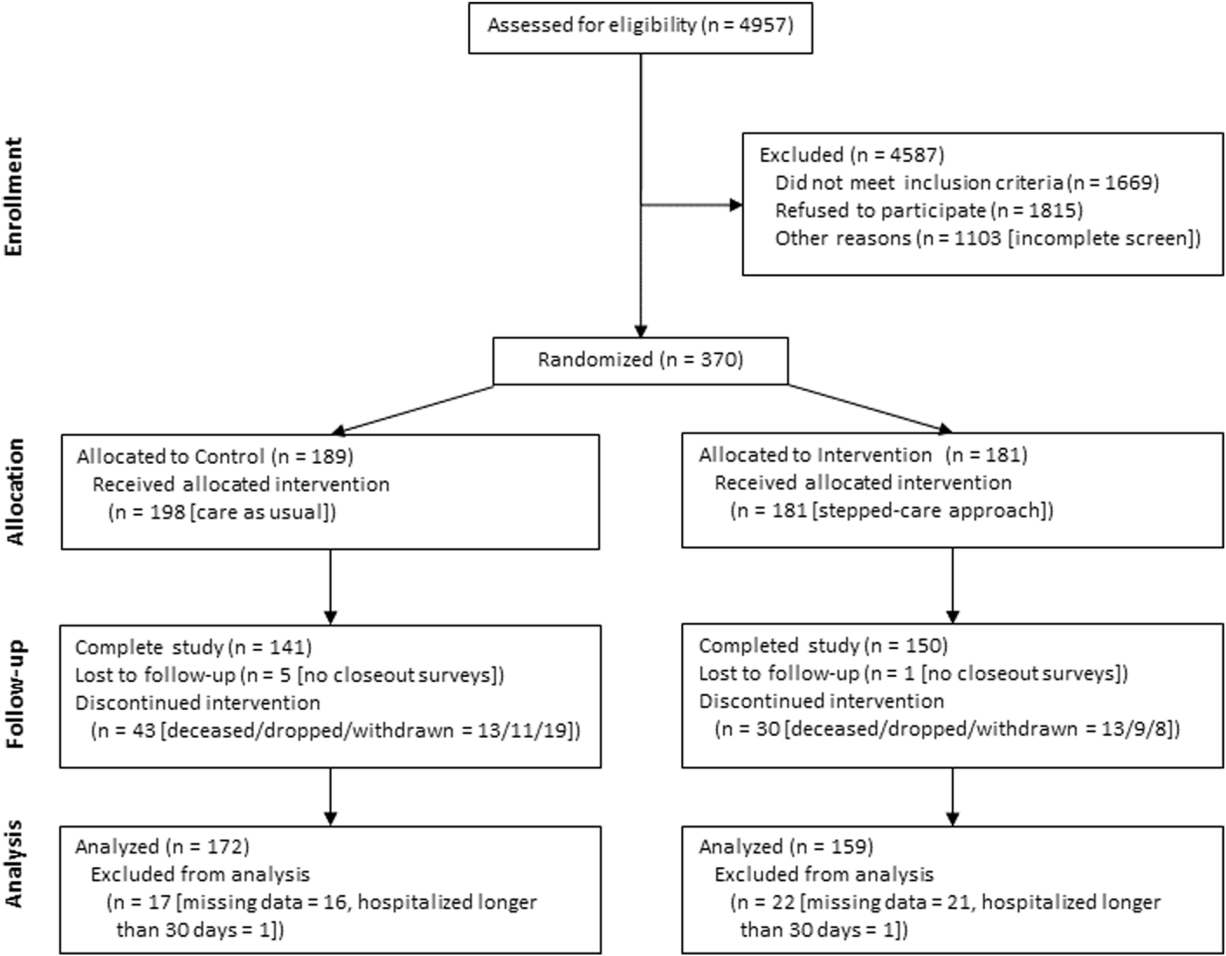
Participant flowchart. Summarizes the recruitment, randomization, and retention flow of patients in this study, leading to the final analyzed cohort.

The participant flow through the study is illustrated in Fig. 1. In summary, 370 patients were enrolled in the period May 2017 – July 2018 and randomly assigned to either the CG (*n* = 189) or IG (*n* = 181). We excluded patients with missing data and those hospitalized for longer than 30 days, according to one of the exclusion criteria. Hence, there were 172 (91%) and 159 (88%) patients in the CG and IG (total *n* = 331), respectively, included in the intention-to-treat data analysis. Thus, we included healthcare encounters and costs of patients who died, have dropped, were withdrawn, or lost to follow-up in the data analysis for the period they participated in the study. The final patient closed-out in April 2019.

### Baseline characteristics

There were no statistically significant differences between the baseline characteristics of the CG and IG (see Tables 1 and 2). The overall study population (*n* = 331) had median age of 80 years, female (67%), white (85%), widowed (44%), college degree or higher (46%), and retired (87%). The most common comorbidities were hypertension (60%), inflammatory pain disorders (such as arthritis, fibromyalgia, etc., 58%), high cholesterol (36%), and cancer (31%). Nearly two-thirds (62%) of patients reported at least three of the surveyed conditions. Further, there were no statistically significant differences between the healthcare utilization and costs of the CG and IG during the 90-day observation period prior to the 180-day intervention period.

**Table 1:** Baseline characteristics by group—demographics.

Baseline characteristics	Population *n* = 331	Control *n* = 172	Intervention *n* = 159	*p* value
Study Status, *n* (%)				0.198
Closed Out	258 (77.9)	129 (75.0)	129 (81.1)	
Withdrawn	27 (8.2)	19 (11.0)	8 (5.0)	
Deceased	26 (7.9)	13 (7.6)	13 (8.2)	
Dropped	20 (6.0)	11 (6.4)	9 (5.7)	
Existing Lifeline subscribers, *n* (%)	112 (33.6)	53 (30.6)	59 (36.9)	0.277
Gender, Male, *n* (%)	108 (32.6)	57 (33.1)	51 (32.1)	0.929
Age, *median years (IQR)*	80 (74, 86)	80 (74, 86)	81 (74, 87)	0.441
Race, *n* (%)				0.601
White	282 (85.2)	142 (82.6)	140 (88.1)	
Black or African American	27 (8.2)	18 (10.5)	9 (5.7)	
Asian	7 (2.1)	4 (2.3)	3 (1.9)	
Hispanic or Latino (any race)	6 (1.8)	4 (2.3)	2 (1.3)	
Other or more than one race	6 (1.8)	3 (1.7)	3 (1.9)	
Unknown	3 (0.9)	1 (0.6)	2 (1.3)	
Marital status, *n* (%)				0.239
Widowed	146 (44.1)	79 (45.9)	67 (42.1)	
Married or partnered	101 (30.5)	47 (27.3)	54 (34.0)	
Divorced or separated	45 (13.6)	26 (15.1)	19 (11.9)	
Single, never been married	36 (10.9)	20 (11.6)	16 (10.1)	
Other or unknown	3 (0.9)	0 (0.0)	3 (1.9)	
Living with someone (vs. alone), *n* (%)	168 (50.8)	80 (46.5)	88 (55.3)	0.135
Educational level, *n* (%)				0.424
Less than high school	22 (6.6)	13 (7.6)	9 (5.7)	
High school or GED	84 (25.4)	45 (26.2)	39 (24.5)	
Some college or vocational/technical training	71 (21.5)	42 (24.4)	29 (18.2)	
College graduate	69 (20.8)	31 (18.0)	38 (23.9)	
Post-graduate degree	84 (25.4)	40 (23.3)	44 (27.7)	
Other or unknown	1 (0.3)	1 (0.6)	0 (0.0)	
Employment status, *n* (%)				0.664
Retired	288 (87.0)	154 (89.5)	134 (84.3)	
Disabled	15 (4.5)	5 (2.9)	10 (6.3)	
Employed	14 (4.2)	6 (3.5)	8 (5.0)	
Homemaker	5 (1.5)	3 (1.7)	2 (1.3)	
Unemployed	4 (1.2)	2 (1.2)	2 (1.3)	
Other or unknown	5 (1.5)	2 (1.2)	3 (1.9)

**Table 2:** Baseline characteristics by group—comorbidities.

Baseline characteristics	Population *n* = 331	Control *n* = 172	Intervention *n* = 159	*p* value
Comorbidities*, n* (*%*)				
Hypertension	199 (60.1)	104 (60.5)	95 (59.7)	0.983
Inflammatory pain disorders	191 (57.7)	101 (58.7)	90 (56.6)	0.781
High cholesterol	120 (36.3)	55 (32.0)	65 (40.9)	0.117
Cancer	101 (30.5)	54 (31.4)	47 (29.6)	0.808
Chronic heart disease	72 (21.8)	37 (21.5)	35 (22.0)	1.000
Diabetes	66 (19.9)	40 (23.3)	26 (16.4)	0.152
Depression	60 (18.1)	31 (18.0)	29 (18.2)	1.000
Chronic obstructive pulmonary disorder	53 (16.0)	30 (17.4)	23 (14.5)	0.557
Asthma	49 (14.8)	25 (14.5)	24 (15.1)	1.000
Stroke	50 (15.1)	29 (16.9)	21 (13.2)	0.439
Congestive heart failure	43 (13.0)	24 (14.0)	19 (11.9)	0.705
Acute myocardial infarction	43 (13.0)	27 (15.7)	16 (10.1)	0.174
Other	45 (13.6)	20 (11.6)	25 (15.7)	0.355
None	13 (3.9)	7 (4.1)	6 (3.8)	1.000
Total number of comorbidities, *n* (%)				0.867
0	13 (3.9)	7 (4.1)	6 (3.8)	
1	43 (13.0)	22 (12.8)	21 (13.2)	
2	72 (21.8)	36 (20.9)	36 (22.6)	
3	77 (23.3)	37 (21.5)	40 (25.2)	
≥4	126 (38.1)	70 (40.7)	56 (35.2)	

To select features for inclusion in healthcare cost linear regression, we performed sensitivity analyses using ANOVA to compare baseline models (study group and intervention duration only as independent variables) with models adjusted for patient demographics such as age, gender, race, and living situation. None of the ANOVA results comparing adjusted vs. baseline models crossed the *p* < 0.05 significance threshold, suggesting that the baseline models are sufficient and thus are presented in the results section below.

### Clinical outcomes

The clinical outcomes results are summarized here and described in detail in^7^. First, the primary outcome of ED encounters rate was not statistically significant (15% decrease, *p* = 0.291). Second, the intervention group (IG) had 68% fewer 90-day readmissions (*p* = 0.007) compared to the control group (CG) with a corresponding 76% reduction in the proportion of patients with 90-day readmission (9.9% control vs. 2.5% intervention group, *p* = 0.011). Third, the IG had 53% fewer 180-day readmissions (*p* = 0.020) compared to the CG. Fourth, the EMS utilization was less in the IG compared with the CG—IG had 49% fewer EMS encounters (*p* = 0.006) compared to the CG. Finally, other outcomes that decreased in the IG compared to the CG but did not reach statistical significance include inpatient encounters (14% decrease, *p* = 0.438), 30-day readmission rates (57% decrease, *p* = 0.083), and number of ED transports (33% decrease, *p* = 0.153). Below we describe the inpatient and outpatient encounters that underpin the healthcare cost results.

### Inpatient and outpatient encounters

There were 37 patients (21.5%) having a total of 58 inpatient encounters in the CG vs. 37 patients (23.3%) with a total of 46 inpatient encounters in the IG. The inpatient encounters rate in the IG was 14% lower than the CG (regression model coefficient: 0.86, 95% CI = (0.58, 1.26)), however, it was not statistically significant (*p* = 0.438). There were 125 patients (72.7%) having a total of 924 outpatient encounters in the CG vs. 119 patients (74.8%) with a total of 922 outpatient encounters in the IG. The outpatient encounters rate in the IG was 8% higher than the CG (regression model coefficient: 1.08, 95% CI = (0.99, 1.18)), however, it was not statistically significant (*p* = 0.101).

**Table 3:** Summary of healthcare costs in both groups during the intervention period.

Healthcare cost	Inpatient cost, $		Outpatient cost, $		Total cost, $	
Intervention period	CG*	IG**	CG	IG	CG	IG
30-day	$139,175	$97,468	$123,642	$67,556	$262,817	$165,024
60-day	$254,725	$193,510	$231,601	$128,165	$486,326	$321,675
90-day	$413,156	$247,350	$334,291	$223,235	$747,447	$470,585
120-day	$500,853	$361,620	$390,415	$293,289	$891,268	$654,909
150-day	$832,121	$524,632	$446,385	$372,629	$1,278,506	$897,261
180-day	$1,021,437	$656,188	$526,918	$485,902	$1,548,355	$1,142,090
Cost per patient, $ mean (sd)	$5,939 ($ 16,962)	$4,127 ($9,503)	$3,063 ($8,993)	$3,056 ($7,716)	$9,002 ($22,047)	$7,182 ($13,304)

*CG Control Group with 172 patients, **IG Intervention Group with 159 patients

### Control group healthcare costs

**Fig. 2:**
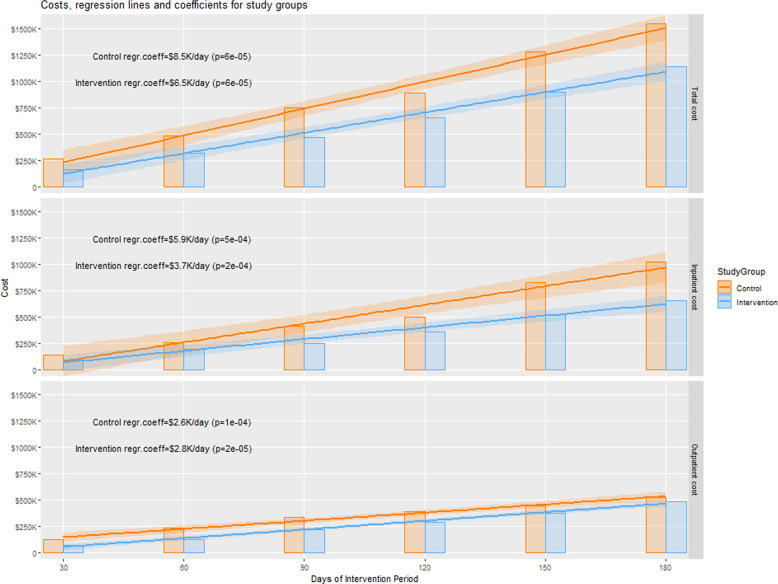
Healthcare cost results within each group. Illustrates the regression lines and coefficients (lines slopes) for each study group. The regression coefficients indicate the expected daily cost increase in each group for the accumulated total, inpatient and outpatient healthcare costs in the top, middle, and bottom panels, respectively.

**Fig. 3:**
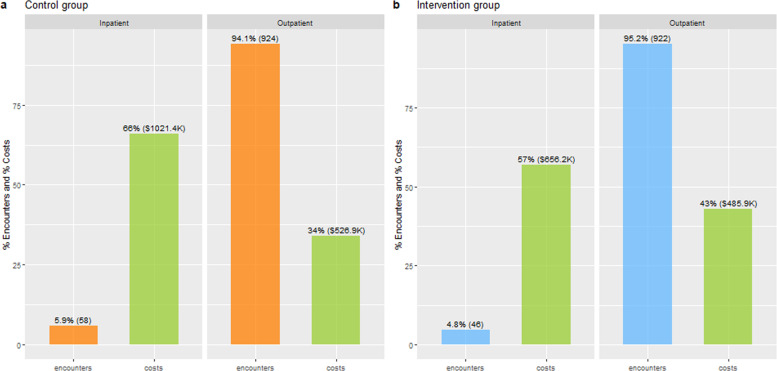
Distributions of the inpatient and outpatient encounters and their costs in the control group (**a**) and the intervention group (**b**).

The total costs for inpatient and outpatient encounters in the CG was $262,817 in the 1st month and accumulated to $1,548,355 in the intervention period (6 months), as illustrated in Fig. 2 and Table 3. The average total cost per patient was $9,002 (SD $22,047). About 66% of the total cost of the CG included inpatient encounter costs, see Fig. 3, which accumulated from $139,175 in the 1st month to $1,021,437 by the 6th month of the intervention period, see Table 3. The average inpatient encounter cost per patient was $5,939 (SD $16,962). The remaining 34% of the total cost of the CG was outpatient encounters costs, which accumulated from $123,642 in the 1st month to $526,918 by the 6th month of the intervention period. The average outpatient encounters cost per patient was $3,063 (SD $8,993).

Inferential analysis of CG costs based on linear regression showed that the average daily increase in total healthcare costs of the CG was $8.5 K and it is statistically significant (*p* < 0.001), see Fig. 2. This growth was primarily driven by a significant increase of $5.9 K (*p* < 0.001) in the average daily cost of the inpatient encounters, followed by an increase of $2.6 K (*p* < 0.001) in the average daily cost of the outpatient encounters.

### Intervention group healthcare costs

The total cost of inpatient and outpatient encounters in the IG was $165,024 in the 1st month and accumulated to $1,142,090 in the intervention period, as illustrated in Fig. 2 and Table 3. The average total cost per patient was $7,182 (SD $13,304). About 57% of the total cost of the IG included inpatient encounter costs, see Fig. 3, which accumulated from $97,468 in the 1st month to $656,188 by the 6th month of the intervention period. The average inpatient encounter cost per patient was $4,127 (SD $9,503). The remaining 43% of the total cost of the IG was outpatient encounter costs, which accumulated from $67,556 in the 1st month to $485,902 by the 6th month of the intervention period. The average outpatient encounter cost per patient was $3,056 (SD $7,716).

Inferential analysis of IG costs based on linear regression showed that the average daily increase in total healthcare costs of the IG was $6.5 K and it is statistically significant (*p* < 0.001), see Fig. 2. This growth was primarily driven by a significant increase of $3.7 K (*p* < 0.001) in average daily cost of the inpatient encounters, followed by an increase of $2.8 K (*p* < 0.001) in the average daily cost of the outpatient encounters.

### Between-groups healthcare costs comparison

Analysis of both CG and IG total healthcare costs showed a daily total cost reduction of $2,049 in the IG compared to the CG, (regression model coefficient CG: $8,522, 95% CI = ($7,539, $9,505), *p* < 0.001, and IG: -$2,049, 95% CI = (−$3,440, −$659), *p* = 0.0094), as depicted in Table 4. To account for the difference in group size, a linear regression analysis of the total encounter costs per patient was performed as well. The daily total cost per patient decreased by $9 in the IG compared to the CG, (regression model coefficient CG: $50, 95% CI = ($44, $55), *p* < 0.001, and IG: −$9, 95% CI = (−$17, −$0.5), *p* = 0.0402). Similar analyses were performed for both the inpatient encounter costs and the outpatient encounter costs. The model coefficients for each study group and the corresponding *p* values are listed in Table 4.

**Table 4:** Healthcare cost regression results between groups.

Healthcare costs	CG, *n* = 172	IG, *n* = 159	Model Coef* (95% CI)	*p* value**
Inpatient cost***, $	$1,021,437	$656,188	CG: 5,935 (4,872, 6,997)	1.25e−06
			IG: −2,219 (−3,722, −716)	0.0093
Inpatient cost per patient***, $ mean (sd)	$5,939 ($ 16,962)	$4,127 ($9,503)	CG_pt: 35 (28, 41)	1.43e−06
			IG_pt: −11 (−20, −2)	0.0203
Annualized inpatient cost per patient, $ (95% CI)	$11,861 ($10,046, $13,679)	$8,140 ($6,323, $9,957)	Reduction: $3.7 K (−31%)	0.0203
Outpatient cost***, $	$526,918	$485,902	CG: 2,588 (2,247, 2,928)	1.14e−07
			IG: + 170 (−311, 651)	0.4390
Outpatient cost per patient***, $ mean (sd)	$3,063 ($8,993)	$3,056 ($7,716)	CG_pt: 15 (13, 17)	1.40e−07
			IG_pt: +2 (−0.6, 5)	0.1013
Annualized outpatient cost per patient, $ (95% CI)	$5,826 ($5,238, $6,411)	$6,069 ($5,483, $6,655)	Increase: $243 (+4%)	0.1013
Total cost***, $	$1,548,355	$1,142,090	CG: 8,522 (7,539, 9,505)	4.09e−08
			IG: −2,049 (−3,440, −659)	0.0094
Total cost per patient***, $ mean (sd)	$9,002 ($22,047)	$7,182 ($13,304)	CG_pt: 50 (44, 55)	5.17e−08
			IG_pt: −9 (−17, −0.5)	0.0402
Annualized total cost per patient, $ (95% CI)	$17,687 ($15,986, $19,387)	$14,209 ($12,509, $15,910)	Reduction: $3.5 K (−20%)	0.0402

*The IG model coefficient indicates decrease(−)/increase(+) in $ compared to the CG model coefficient.

**p values of Linear regression models.

***Costs at the end of the 180-day intervention period.

Finally, the linear regression models used for inferential analysis were also used to calculate the annualized cost per patient in each group. In summary, the annualized total cost reduction per patient in the IG vs. CG was 20% (3.5 K, $$17.7\,{\mathrm{K}}\,vs.$$ 14.2 K, *p* = 0.04). It was primarily driven by 31% lower annualized inpatient cost per patient of the IG compared with the CG (3.7 K, $$8.1\,{\mathrm{K}}\,vs.$$ 11.8 K, *p* = 0.02). The annualized outpatient cost per patient was 4% higher in the IG compared to the CG ($$243$$, 6.1 K vs. $5.8 K, *p* = 0.10). However, this finding was not statistically significant.

### Inpatient and outpatient encounters and their cost

The CG had 58 (5.9%) inpatient and 924 (94.1%) outpatient encounters as illustrated in Fig. 3. The 6% inpatient encounters constituted 66% ($1,021.4 K) of the total healthcare cost of the CG and the average cost per inpatient encounter was $17,611 (SD $16,241). In contrast, the 94% outpatient encounters constituted only 34% ($526.9 K) of the total healthcare cost of the CG and the average cost per outpatient encounter was $570 (SD $1,237). The IG had similar proportions of inpatient and outpatient encounters to the CG—46 (4.8%) inpatient and 922 (95.2%) outpatient encounters as illustrated in Fig. 3. The 5% inpatient encounters constituted 57% ($656.2 K) of the total healthcare cost of the IG and the average cost per inpatient encounter was $ 14,265 (SD $11,116). In contrast, the 95% outpatient encounters constituted 43% ($485.9 K) of the total healthcare cost of the IG and the average cost per outpatient encounter was $527 (SD $1,378).

### Patients with single vs. multiple inpatient encounters

**Fig. 4:**
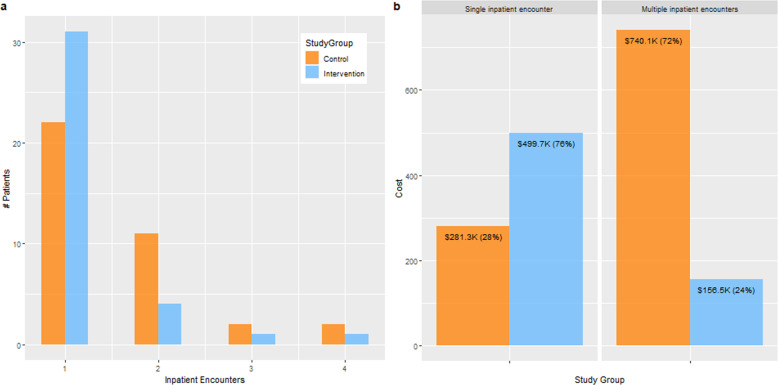
Patients with single vs. multiple inpatient encounters and their costs. Summarizes between-groups differences in (**a**) patients with single and multiple inpatient encounters and (**b**) associated costs.

Figure 4 illustrates the (a) inpatient encounters and (b) their cost by comparing the patients with single vs. multiple events in the CG and IG. The total number of patients having inpatient encounters was comparable in both groups—37 (21.5%) in the CG vs. 37 (23.3%) in the IG, *p* = 0.801. However, the proportions of patients having single vs. multiple inpatients encounters in both groups were almost statistically different (*p* = 0.063). There were 22 (12.8%) patients in the CG vs. 31 (19.5%) in the IG having a single inpatient encounter during the intervention period. There was a >50% decrease in patients with multiple inpatient encounters in the CG vs. the IG (15 (8.7%) vs. 6 (3.8%)). Also, the corresponding inpatient encounters were fewer in the IG vs. the CG (15 vs. 36). They accounted for 72% ($740.1 K out of $1021.4 K) of inpatients encounters cost in the CG vs. only 24% ($156.5 K out of $656.2 K) in the IG, see Fig. 4.

### Cost analyses by ICD-10 diagnosis categories

We used Clinical Classifications Software Refined (CCSR) to group each inpatient encounter’s primary ICD-10 diagnosis code into categories for a subgroup analysis. Diagnostic categories were then ordered by most to least expensive, and we selected the top 10 costliest diagnosis categories to summarize overall and according to (1) study group assignment, and (2) among patients with single or multiple inpatient encounters. Table 5 presents these data. Each cell represents the number of inpatient encounters associated with each diagnosis category with the average cost per encounter in parentheses. There is category diversity both between groups and between patients with single vs. multiple inpatient encounters with exception of Heart Failure, which was common among both study groups. Overall, inpatient encounters with Non-rheumatic and unspecified valve disorders, Heart failure, Septicemia, or Gastrointestinal hemorrhage as the primary diagnosis category contributed to roughly a quarter (27.4%) of the total inpatient costs during the intervention period, and the top 10 costliest categories alone (out of 62 categories present in the data) contributed to roughly half of the costs (46.7%).

**Table 5:** Top 10 most expensive inpatient encounters grouped by diagnostic categories.

Patients with inpatient encounters	Single encounter			Multiple encounter		
CCSR Diagnostic Category, *n* ($ mean cost)	CG	IG	Overall	CG	IG	Overall
Nonrheumatic and unspecified valve disorders				2 ($73,659)*		2 ($73,659)
Heart failure^CG, IG^^**^	3 ($10,660)	2 ($12,383)	5 ($11,349)	4 ($9,979)	3 ($8,839)	7 ($9,491)
Septicemia^CG^				3 ($31,505)		3 ($31,505)
Gastrointestinal hemorrhage^IG^		1 ($40,958)	1 ($40,958)	2 ($7,582)	1 ($38,007)	3 ($17,724)
COPD and bronchiectasis^CG^	3 ($9,175)		3 ($9,175)	2 ($19,444)		2 ($19,444)
Biliary tract disease^CG^	1 ($20,300)		1 ($20,300)	2 ($19,448)		2 ($19,448)
Pneumonia (except that caused by tuberculosis)	1 ($16,886)	1 ($15,750)	2 ($16,318)	1 ($21,905)		1 ($21,905)
Cardiac dysrhythmias		2 ($25,644)	2 ($25,644)			
Other specified nervous system disorders^CG^	1 ($27,908)		1 ($27,908)	2 ($9,810)		2 ($9,810)
Abdominal hernia				1 ($45,795)		1 ($45,795)

*Each cell represents the number of inpatient encounters associated with each diagnosis category with the average cost per encounter in parentheses.

**CG, IG—among top 10 most frequent conditions for the Control and the Intervention group.

## Discussion

The analyses revealed three key findings. First, the Stepped-Care intervention reduced the annualized total healthcare cost per patient in the IG compared to the CG by $3.5 K (20%, $14.2 K vs. $17.7 K, *p* = 0.04). This reduction was driven by the $3.7 K lower annualized inpatient costs per patient in the IG compared to the CG (31%, $8.1 K vs. $11.8 K, *p* = 0.02) as both groups had similar annualized outpatient cost per patient ($6.1 K vs. $5.8 K, *p* = 0.10). In the published literature, there are several interventions shown to improve the quality of care provided to older patients with multiple chronic conditions, including the Guided Care Model^14^, Project BOOST (Better Outcomes for Older adults through Safe Transitions)^15^, the Transitional Care Model^16^, and Mobile Integrated Healthcare^17^. The Guided Care model demonstrated an annual net savings of $1364 per patient in comparison to care-as-usual, while the Transitional Care Model showed cost reductions of up to $4027 in cognitively impaired older adults, 180 days post-hospital discharge.

The second key finding is that inpatient encounters were a small proportion of all encounters in both groups—5.9% in the CG vs. 4.8% in the IG. However, they contributed to 66% of the total costs in the CG and 57% of the total in the IG. This finding of disproportion between the number of encounters and their costs is consistent with the results of an analysis performed by Centers for Medicare and Medicaid Services (CMS)^18^ and American Hospital Association (AHA)^19^. The Stepped-Care intervention has the potential to reduce this disproportion.

The third key finding is that the significant reduction in the number of inpatient encounters in the IG was in subgroup of patients with multiple hospitalizations, i.e., the high utilizers. They accounted for almost 3/4 of inpatients encounters cost in the CG vs. only 1/4 in the IG, see Fig. 4. Thus, the Stepped-Care intervention was most effective in reducing inpatient encounters and associated costs of the high utilizers in the middle segment of the cost pyramid. Therefore, this study provides clinical evidence on interventions effectiveness for patients in the middle segment of the cost pyramid while several previously published studies have focused on demonstrating the effectiveness of care programs for super utilizers in the top segment of the cost pyramid^20–22^.

In 2020, the COVID-19 pandemic prompted a large acceleration in digital medicine^23^, with the demand for telehealth rising dramatically as the pandemic continues to disrupt the practice of medicine and the delivery of healthcare worldwide^24^. Many HCOs have turned to virtual visits and remote patient monitoring to continue caring for patients while minimizing the risk of virus transmission and reducing the strain on scarce hospital resources. New models of team-based digital medicine that blend the best elements of traditional and virtual healthcare are emerging^25,26^, and the Stepped-Care intervention is an example of such a team-based approach. These newer care management programs are of great importance for the most vulnerable patients—the older adults—who can benefit greatly from avoiding unnecessary visits to the hospital especially during a pandemic. Therefore, innovative population health management programs that leverage telehealth such as PERS and a Stepped-Care intervention, have significant potential to improve clinical outcomes, drive down associated healthcare costs and help HCOs achieve the quadruple aim. Further, the recent changes in regulations and payment policies made by the CMS and some commercial payers in response to COVID-19 have accelerated the growth of technology-based tools and interventions for older patients^27,28^, facilitating the implementation of care management programs.

This study has some limitations. First, as shown in Table 1, the study population is mostly older, primarily female, white, living alone, and highly educated, thus limiting the generalizability of the study. Second, the clinical encounters and costs not captured in the medical records or in the claims data of MGB were unavailable to analyze. Therefore, our analyses do not include costs of inpatient or outpatient encounters that happened outside of the MGB system. All these limitations may have affected the magnitude of the cost reduction in the IG and CG. Despite the limitations, the study provides valuable evidence for the effectiveness of the Stepped-Care intervention in reducing healthcare utilization and associated costs in older patients with multiple conditions.

In conclusion, this randomized controlled trial highlights the impact of telehealth-based population health management programs on improving clinical outcomes and reducing healthcare costs in older adults in the middle segment of the cost pyramid. The Stepped-Care intervention used in this study, which combines actionable predictive analytics and tailored interventions, has great potential to support HCOs in the post-COVID era to provide high-quality care in home and community settings, alleviating the pressure on resource-constrained healthcare systems.

## Methods

The methods used in the study are summarized according to CONSORT (Consolidated Standards of Reporting Trials) recommendations^29^. A full description of the study protocol is available in^6^.

### Design

The study was implemented as a two-arm randomized controlled trial. All enrolled patients were randomized by the study coordinator via a computerized random-number generator to either an IG or a CG. Treatment allocation was concealed in an opaque envelope opened after the informed consent procedures, so patients and the enrolling study staff were blinded to allocation until then.

The study period was 9 months in total, consisting of a 3-month observation period followed by a 6-month intervention period. The observation period was necessary to collect PERS data from patients who are new to the PERS service to make accurate predictions using the predictive model. Enrollment began in May 2017 and stopped upon reaching the target of 370 patients in July 2018 (determination of sample size described under Statistical Analysis below).

The study was approved by the MGB Human Research Committee, the Institutional Review Board for the MGB and registered as NCT03126565 at ClinicalTrials.gov, first posted on April 24th, 2017^30^.

### Participants

Study participants were identified among MGB patients who received home care from PHH to manage their chronic conditions. Eligible patients were 65 years or older and English-speaking. Further, PHH patients in the middle segment of the cost pyramid were identified and selected for the study. Middle segment means that the total healthcare costs were within the 6th − 50th percentile of the cost pyramid for the fiscal year prior to their enrollment (2016 for patients enrolled in 2017; 2017 for patients enrolled in 2018). The middle segment cutoffs for 2016 and 2017 were projected based on 2011–2015 data from the retrospective analysis that was conducted prior to this prospective study^13^. Patients with implanted devices (as a precautionary measure against possible interference with PERS) and those suffering from dementia, Alzheimer’s, or psychiatric illness (anxiety disorder or psychosis) were excluded from the study. Patients with an inpatient encounter resulting in a length-of-stay longer than a month (1/6 of the intervention period) or discharged into long-term Skilled Nursing Facilities were also excluded from the study. Finally, patients missing identifiers necessary to map the patient’s EHR and PERS data, thus preventing the predictive model risk score calculation, were excluded as well, since the junction in the Stepped-Care intervention at which the study nurse would contact a high-risk IG patient was precluded. Exclusion criteria were applied regardless of the group assignment. All patients provided informed written consent prior to participating in the study.

### Predictive model

The predictive model identifying patients at risk of ED transports was used in the first step of the Stepped-Care intervention. The model included three categories of predictors as input: (1) PERS utilization data (e.g., frequency and recency of various types of incidents at home, such as falls, respiratory issues, and chest pain) and incidents outcomes (e.g., responder assistance, EMS assistance, EMS transport to ED), (2) self-reported medical conditions provided at PERS enrollment (e.g., high blood pressure, diabetes, congestive heart failure), and (3) other factors such as age, gender, and time on the PERS service. The predictive model had an Area under the Receiver Operator Characteristic Curve (AUC) of 78%. As a part of the model validation, the model’s predictions were compared with clinical outcomes derived from the MGB electronic health record. One of the findings was that patients who were predicted to be high-risk had nearly four times higher rates of ED encounters than the low-risk patients^5^.

### Intervention

All patients received a PERS at home, connecting them to a response center any time they needed help during the study period. In addition, all participants were instructed to directly push the PERS help button or call 911 for connection to EMS if they experienced worsening of symptoms or required immediate attention.

**Fig. 5:**
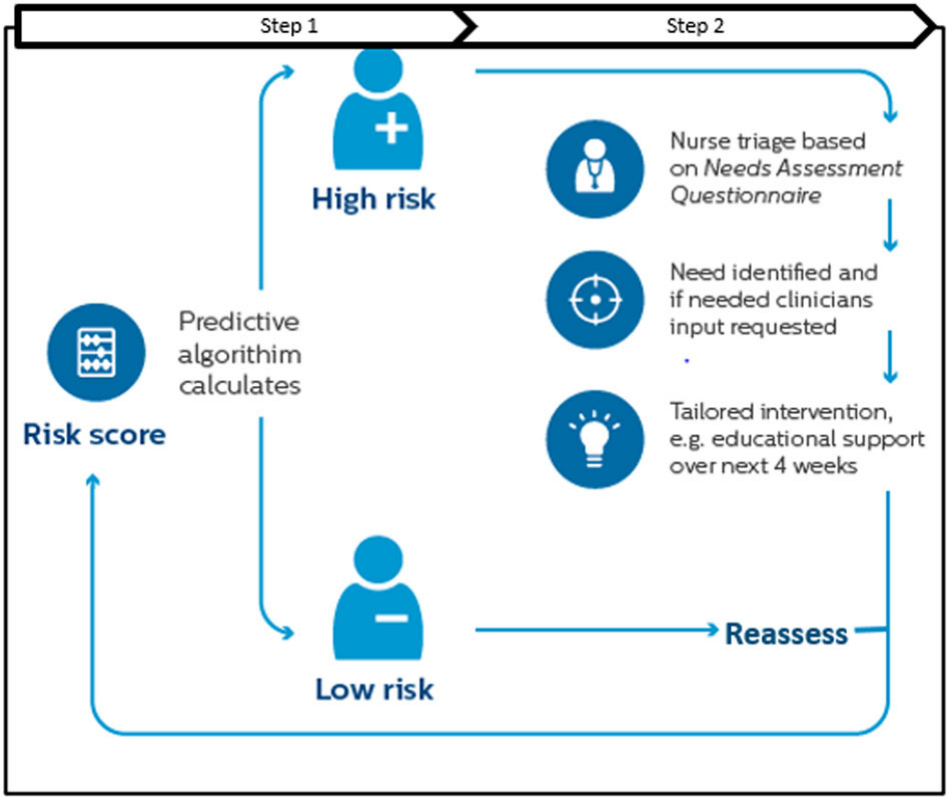
Intervention design—stepped-care intervention. Shows the (step one) predictive model output as risk scores, followed by (step two) nurse triage for patients flagged as high risk. The figure also demonstrates how patients are regularly reassessed by the model.

During the initial 3-month observation period, the patients from both the CG and the IG continued to receive care-as-usual from their care providers and no study interventions were administered. This was necessary to collect data from the patients new to the PERS service as input to the predictive algorithm. While the CG continued to receive care-as-usual during the subsequent 6-month intervention period, the IG received care according to the Stepped-Care intervention depicted in Fig. 5. In Step 1 of the intervention, each IG patient’s risk of ED transport in the upcoming 30-days was assessed daily by the predictive model, starting on the first day of their 6-month intervention period. The study nurse reviewed the risk score dashboard and for high-risk patients initiated Step 2 of the Stepped-Care intervention, namely a tailored intervention. It always started with a triage, during which the study nurse completed a needs assessment questionnaire with the patient via phone. The assessment included general health questions as well as specific questions on respiratory symptoms, physical activity, activity of daily living, pain, and bladder control^6^. Based on the patient’s needs and clinician’s input (if needed), a care plan was assigned. The study population consisted of a clinically diverse patient cohort and hence, no single tailored plan was designated prior to the study but rather several care plan components—such as follow-up reassessment via phone including tailored feedback, patient education over a 4-week period, home visits, or outpatient visits to primary care physician, or telemonitoring—were assigned to the patients by the nurse. Thus, the study aims to evaluate the combined effect of both steps of the Stepped-Care intervention on healthcare utilization and costs, rather than a single pre-defined care plan.

### Healthcare cost measurements

The total healthcare cost in this study was defined as a sum of the cost of all inpatient and outpatient encounters. The cost of each inpatient or outpatient encounter is a sum of variable and fixed costs for direct and indirect patient care. Inpatient encounters were defined as hospital admissions that resulted in a patient being admitted to the hospital for any reason. Outpatient encounters were defined as either ambulatory care or medical or surgical care that did not include an overnight stay. All inpatient and outpatient encounters were counted if they occurred between days 1–180 of the intervention period. All study outcomes measurements are described in^7^.

### Data collection

Patients’ baseline characteristics and needs were collected using enrollment and needs assessment questionnaires developed by the study investigators^6^. These data were recorded in a REDCap database, a secure Web application for building and managing web-based surveys and databases.

All data pertaining to healthcare utilization and costs were extracted from the MGB Enterprise Data Warehouse (EDW), which is a repository of clinical, operational, and hospital cost data of patients receiving care across MGB. Thus, the healthcare costs included billing and internal costs to MGB; it did not refer to insurance payments or costs. Patient data collected were aggregated and analyzed after the last patient closed out of the study and all data were de-identified before analyses.

### Statistical analysis

The sample size for the primary outcome ED encounters was derived based on power analysis with the following parameters: two-armed randomized controlled design with 1:1 allocation ratio and a power of 0.80 at a two-sided significance level of *α* = 0.05 with an intervention effect size of 0.35. This power calculation estimated a sample size of 160 patients per arm, which was adjusted for a loss to follow-up of 15%, leading to the study’s total sample size of 370.

The intention-to-treat approach was used for analyzing data in which all participants were included in the group to which they were assigned, regardless of whether they completed the intervention given to the group. Therefore, patients with missing data in the intervention period due to either mortality or study drop-out, or withdrawal, or loss of follow-up were included in the analysis for the period they participated in the study. All data analysis was performed using the statistical software R, version 3.6.1^31^.

Descriptive statistics of patients’ baseline characteristics were summarized by study group as well as total study population and presented as means and standard deviations (SDs), or frequencies and percentages. Comparisons of normally/non-normally distributed continuous variables by groups were conducted using Student *t* tests/Mann–Whitney *U* tests, respectively. For categorical variables, Pearson Chi-square tests were used to examine the association between the groups.

Inpatient and outpatient encounters (as event-count variables) were modeled using Poisson regression, which is a generalized linear model form of regression analysis used to model count data. Models were controlled for baseline characteristics, which differed between groups if needed.

Healthcare costs were modeled using Linear regression, as several models were built to evaluate the accumulated healthcare cost trends within each study group as well as between the groups. These models built on the cost data during the 6-month intervention period were used to predict the expected annual cost referred to as annualized cost in the paper. Each model provides an estimate of the annualized cost increase/decrease either per group or per patient.

The choice of linear regression for modeling the healthcare costs was based on the following statistical principles. The individual patient’s healthcare cost was exponential and formed a sequence of independent exponential random variables. Therefore, their sum—the accumulated healthcare cost—was a random variable with Gamma distribution. By the central limit theorem, if the number of the variables in the sum is large, then Gamma can be approximated by the normal distribution. To double check this, we analyzed the accumulated healthcare costs with both Gamma and Linear regression models, both of which provided similar results. Finally, linear regression models were selected as they are easier to interpret.

All healthcare cost results are presented in United States Dollars (USD$), followed by their standard distribution (SD).

### Reporting summary

Further information on research design is available in the Nature Research Reporting Summary linked to this article.

## Supplementary information

Reporting Summary

## Data Availability

The data that support the findings of this study are available from MGB but restrictions apply to the availability of these data, which were used under an Institutional Review Board approval, and hence not publicly available. De-identified data are, however, available from the authors upon reasonable request and with permission of the MGB Human Research Committee.

## Code availability

The code that supports the findings of this study is based on standard functions and packages available in R, which is an open-source language and environment for statistical computing. No proprietary code or/and mathematical algorithms are used to perform the analysis.
