# Supplementary material for: A randomized trial examining the effect of predictive analytics and tailored interventions on the cost of care
Source: NPJ Digit Med. 2021 Jun 3;4:92. doi: 10.1038/s41746-021-00449-w (PMC8175712; doi:10.1038/s41746-021-00449-w)
Supplement: Supplementary file 1 — Reporting Summary [file 41746_2021_449_MOESM1_ESM.pdf]

nature research

NPJDIGITALMED-01475
Corresponding author(s): Mariana Nikolova-Simons
Last updated by author(s): Mar 15, 2021

Reporting Summary

Nature Research wishes to improve the reproducibility of the work that we publish. This form provides structure for consistency and transparency in reporting. For further information on Nature Research policies, see our Editorial Policies and the Editorial Policy Checklist.

Statistics

For all statistical analyses, confirm that the following items are present in the figure legend, table legend, main text, or Methods section.

n/a	Confirmed	
☐	☒	The exact sample size (n) for each experimental group/condition, given as a discrete number and unit of measurement
☐	☒	A statement on whether measurements were taken from distinct samples or whether the same sample was measured repeatedly
☐	☒	The statistical test(s) used AND whether they are one- or two-sided<br>*Only common tests should be described solely by name; describe more complex techniques in the Methods section.*
☐	☒	A description of all covariates tested
☐	☒	A description of any assumptions or corrections, such as tests of normality and adjustment for multiple comparisons
☐	☒	A full description of the statistical parameters including central tendency (e.g. means) or other basic estimates (e.g. regression coefficient) AND variation (e.g. standard deviation) or associated estimates of uncertainty (e.g. confidence intervals)
☐	☒	For null hypothesis testing, the test statistic (e.g. F, t, r) with confidence intervals, effect sizes, degrees of freedom and P value noted<br>*Give P values as exact values whenever suitable.*
☒	☐	For Bayesian analysis, information on the choice of priors and Markov chain Monte Carlo settings
☒	☐	For hierarchical and complex designs, identification of the appropriate level for tests and full reporting of outcomes
☒	☐	Estimates of effect sizes (e.g. Cohen's d, Pearson's r), indicating how they were calculated

Our web collection on statistics for biologists contains articles on many of the points above.

Software and code

Policy information about availability of computer code

Data collection	The code that supports the collection of this study data is based on SQL and standard REDCap functioning. No proprietary code or/and mathematical algorithms are used.
Data analysis	The code that supports the findings of this study is based on standard functions and packages available in R, which is an open source language and environment for statistical computing. No proprietary code or/and mathematical algorithms are used.

For manuscripts utilizing custom algorithms or software that are central to the research but not yet described in published literature, software must be made available to editors and reviewers. We strongly encourage code deposition in a community repository (e.g. GitHub). See the Nature Research guidelines for submitting code & software for further information.

Data

Policy information about availability of data

All manuscripts must include a data availability statement. This statement should provide the following information, where applicable:

- Accession codes, unique identifiers, or web links for publicly available datasets
- A list of figures that have associated raw data
- A description of any restrictions on data availability

The data that support the findings of this study are available from Mass General Brigham (MGB) but restrictions apply to the availability of these data, which were used under IRB approval, and so are not publicly available. De-identified data are however available from the authors upon reasonable request and with permission of MGB Human Research Committee.

Field-specific reporting

Please select the one below that is the best fit for your research. If you are not sure, read the appropriate sections before making your selection.

☐ Life sciences ☒ Behavioural & social sciences ☐ Ecological, evolutionary & environmental sciences

For a reference copy of the document with all sections, see nature.com/documents/nr-reporting-summary-flat.pdf

Behavioural & social sciences study design

All studies must disclose on these points even when the disclosure is negative.

Study description	Two-arm randomized controlled trial evaluating the impact of a Stepped-Care intervention (predictive analytics + tailored nurse-driven interventions) on the healthcare cost of older adults.
Research sample	Mass General Brigham (MGB) patients aged 65+ who received care from Partners Healthcare at Home (PHH, a preventative homecare management system that offers general care as well as specialized services to patients within MGB) whose healthcare costs were within the middle (6th to 50th percentile) segment of the cost pyramid for the fiscal year prior to their enrollment. The overall study population had median age of 80 years, and were primarily female (67%), white (85%), widowed (44%), college degree or higher (47%), and retired (87%). This older middle-segment population was chosen to evaluate the impact of an intervention designed to benefit older patients with complex care needs who have not yet entered the top-segment (top 5th percentile) of the cost pyramid.
Sampling strategy	The sample size for the primary outcome was derived based on power analysis with the following parameters: two-armed randomized controlled design with 1:1 allocation ratio and a power of 0.80 at 2-sided significance level of $\alpha=0.05$ with an intervention effect size of 0.35. This power calculation estimated a sample size of 160 patients per arm, which was adjusted for a lost to follow-up of 15%, leading to study total sample size of 370. The choice of intervention effect size of 0.35 was based on hypothesis that the intervention will be effective in reducing potentially avoidable hospitalizations with admission source ED. Our previous retrospective analysis of similar population showed that these avoidable admissions were close to 35%.
Data collection	Patient baseline characteristics and patients' needs were collected using enrollment and needs assessment questionnaires developed by the study investigators. These data were maintained in and extracted from a REDCap database, which is a secure Web application for building and managing web-based surveys and databases. All data pertaining to healthcare utilization were extracted from the MGB Enterprise Data Warehouse (EDW), which is a repository of clinical, operational, and hospital cost data of patients receiving care across MGB and is a source for analytics and reporting used by entities within the MGB. PERS utilization data were collected from the Lifeline PERS database, which constitutes data on EMS encounters (emergency care at home), and ED transports (EMS transport to an ED).
Timing	Enrollment began in May 2017 and stopped upon reaching the target of 370 in July 2018.
Data exclusions	Excluded patients were those with implanted devices; dementia, Alzheimer's or other psychiatric illness (anxiety disorder or psychosis); inpatient admissions resulting in a length-of-stay longer than a month (1/6 of intervention period) or discharged into long-term-care facilities including Skilled Nursing Facilities; and patients missing identifiers necessary to map the patient's EHR and PERS data, thus preventing predictive model risk score calculation, since the junction in the Stepped-Care approach at which the study nurse would contact a high-risk IG patient was precluded. Exclusions were applied regardless of group assignment.
Non-participation	A total of 370 patients were enrolled in the study. After excluding the patients with missing data (16 in the CG and 21 in the IG) and those hospitalized longer than 30 days (1 in each group), there were 172 (91%) and 159 (88%) patients in the CG and IG (total n=331), respectively, included in the intention-to-treat data analysis. Patients who died, were dropped, withdrawn, or lost to follow-up were included in the data analysis with incomplete data, i.e. data only for the period they actively participated in the study.
Randomization	All enrolled patients were randomized by the study coordinator via a computerized random-number generator to either an intervention group (IG) or control group (CG). Treatment allocation was concealed in an opaque envelope opened after the informed consent procedures, so patients and the enrolling study staff were blinded to allocation until then. Patients were randomly assigned to either the CG (n = 189) or IG (n = 181).

Reporting for specific materials, systems and methods

We require information from authors about some types of materials, experimental systems and methods used in many studies. Here, indicate whether each material, system or method listed is relevant to your study. If you are not sure if a list item applies to your research, read the appropriate section before selecting a response.

Materials & experimental systems

n/a	Involved in the study	
☒	☐	Antibodies
☒	☐	Eukaryotic cell lines
☒	☐	Palaeontology and archaeology
☒	☐	Animals and other organisms
☐	☒	Human research participants
☐	☒	Clinical data
☒	☐	Dual use research of concern

Methods

n/a	Involved in the study	
☒	☐	ChIP-seq
☒	☐	Flow cytometry
☒	☐	MRI-based neuroimaging

Human research participants

Policy information about studies involving human research participants

Population characteristics	See above
Recruitment	Patients were identified as potentially meeting study criteria through database query by the PHH study nurse, and further screened for eligibility by study coordinators.
Ethics oversight	Mass General Brigham Institutional Review Board

Note that full information on the approval of the study protocol must also be provided in the manuscript.

Clinical data

Policy information about clinical studies

All manuscripts should comply with the ICMJE guidelines for publication of clinical research and a completed CONSORT checklist must be included with all submissions.

Clinical trial registration	NCT03126565
Study protocol	Palacholla RS, Fischer NC, Agboola S et al. Evaluating the Impact of a Web-Based Risk Assessment System (CareSage) and Tailored Interventions on Health Care Utilization: Protocol for a Randomized Controlled Trial. JMIR Res Protoc 2018;7(5):e10045, doi:10.2196/10045
Data collection	See above
Outcomes	This paper focuses on evaluating healthcare costs associated with healthcare utilization of the control and intervention groups in the study. The impact of the Stepped-Care intervention on healthcare utilization outcomes was presented in a separate manuscript and summarized in the results section of this paper.
